# A Rare Giant Cell Tumor of the Distal Fibula and its Management

**DOI:** 10.7759/cureus.666

**Published:** 2016-07-01

**Authors:** Raju Vaishya, Chirag Kapoor, Paresh Golwala, Amit Kumar Agarwal, Vipul Vijay

**Affiliations:** 1 Orthopaedics, Indraprastha Apollo Hospitals; 2 Orthopaedics, Sumandeep Vidyapeeth, Vadodara, Gujarat

**Keywords:** fibula, giant cell tumour, chemical cauterization, reconstruction, bone graft

## Abstract

Giant Cell Tumour (GCT) of the distal fibula is extremely rare and poses challenges in the surgical management. Wide excision or intralesional curettage, along with adjuvant chemical cauterisation can prevent the recurrence of GCT. The excised bone gap needs reconstruction using tricortical iliac autograft and supportive plate fixation. In addition to wide excision, preservation of ankle mortise is advisable in locally aggressive and large lesions of the distal fibula. We report a GCT of the distal fibula in a young female patient. As part of the treatment, en bloc resection, chemical cauterisation with phenol, and distal fibula reconstruction with a tricortical iliac crest bone graft was done. Eighteen months after the treatment, the patient has no recurrence and her ankle is stable with full range of movement. We suggest this method to be worthwhile for the treatment of this uncommon lesion in quantifying recurrence and functional outcome.

## Introduction

The ankle joint is a complex and formed by lower end fibula, tibia, and talus along with complex ligamentous restraints. Lower end fibula forms an important component of ankle mortise. Any pathological lesion or ensuing deformity in lower fibula can lead to an unstable ankle resulting into lifelong debility. The management of any lesion in the lower end of fibula needs to address both the primary pathology management and restoration of the ankle joint for an adequate functional outcome. Giant Cell Tumour (GCT) is one of the most commonly encountered bone tumours in clinical practice. More than 50% of these tumors occur around the knee joint [1]. The upper end of the fibula is more commonly involved, i.e. 2.5 % greater than the distal fibula [2]. The reported incidence of distal fibular involvement is less than 1% of all GCTs [3]. We present a young female with GCT of distal fibula and was managed using an innovative and novel technique.

## Case presentation

An 18-year-old female patient was presented with a slowly growing, painless swelling at the lateral aspect of left ankle since four months. There was no other significant contributing history. Local examination revealed a well-defined bony hard swelling (7 cm x 4 cm) which was nonadherent to the overlying skin. The ankle and subtalar joints had full range of painless movements.

**Figure 1:**
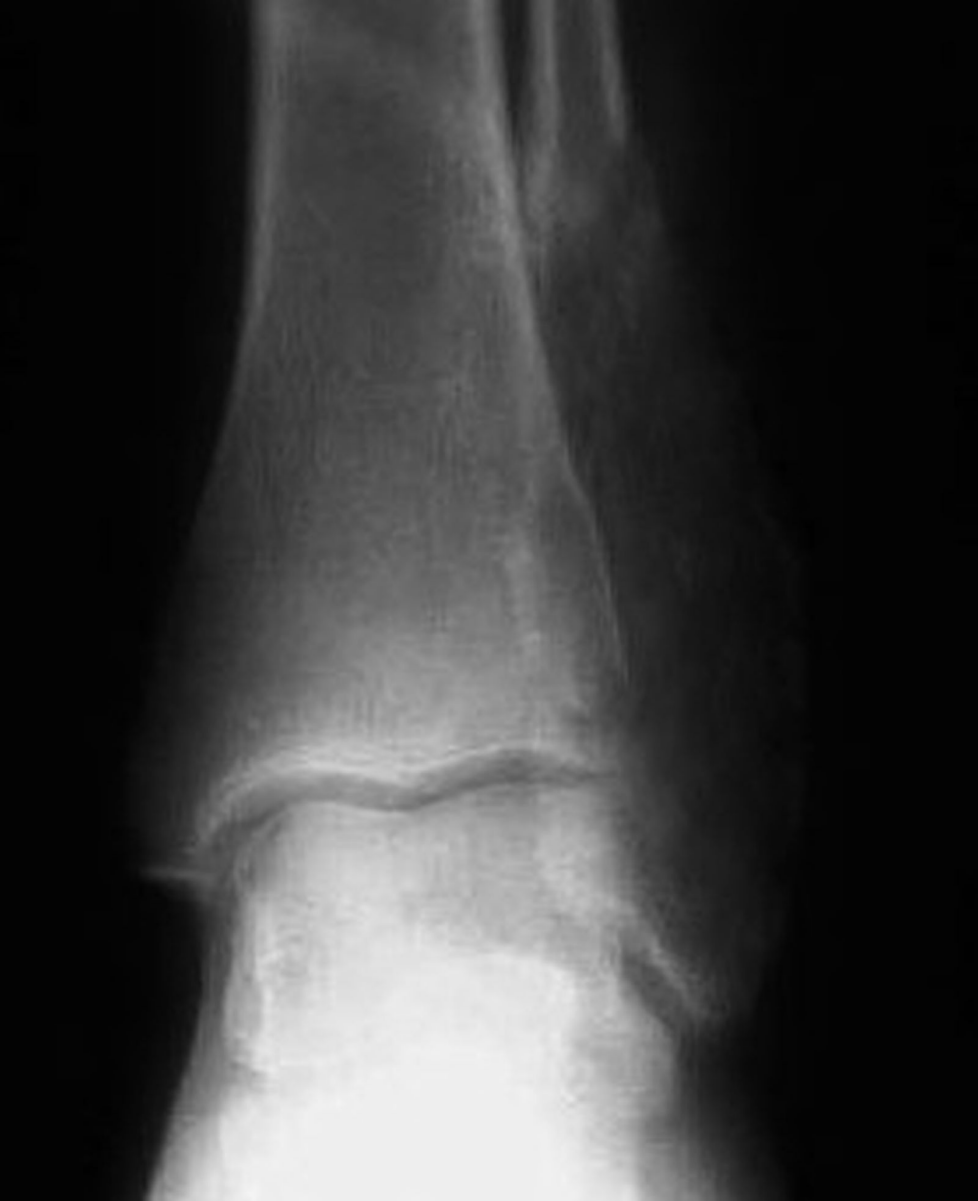
Plain radiograph anteroposterior view showing large expansile lytic lesion involving the distal end of fibula

Plain radiographs revealed a multi-loculated, expansile and well-defined lesion involving the lower end of fibula mimicking a soap bubble like appearance (Figure 1).

**Figure 2:**
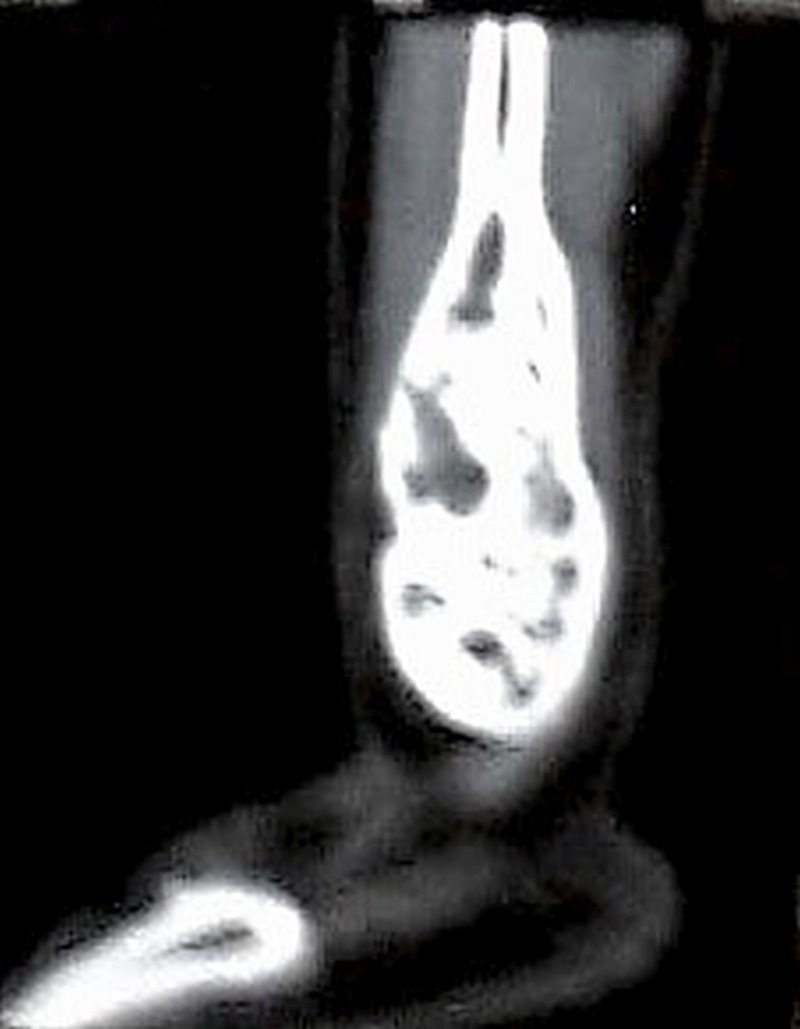
Computed tomography (CT scan)- Sagittal view showing large lesion in distal end of fibula

The radiographic findings were suggestive of a GCT. A Computed Tomogram (CT) confirmed the presence of a large lesion in the distal fibula with no cortical breach with preserved ankle mortise (Figure 2).

**Figure 3:**
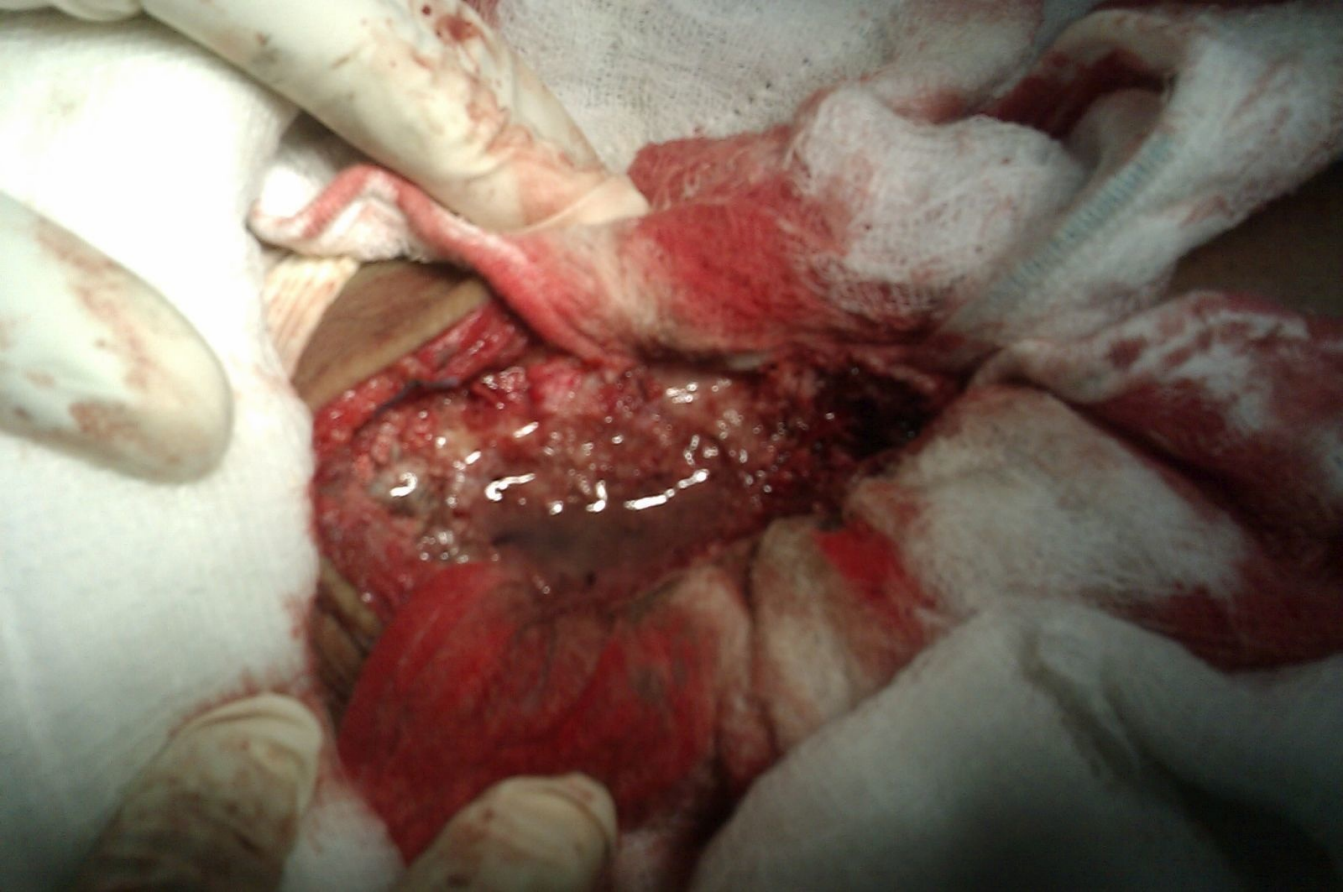
Intra-operative picture showing chemical cauterisation using phenol after curettage of the tumor

An extensive curettage of the tumour along with chemical cauterization was done using phenol (Figure 3).

**Figure 4:**
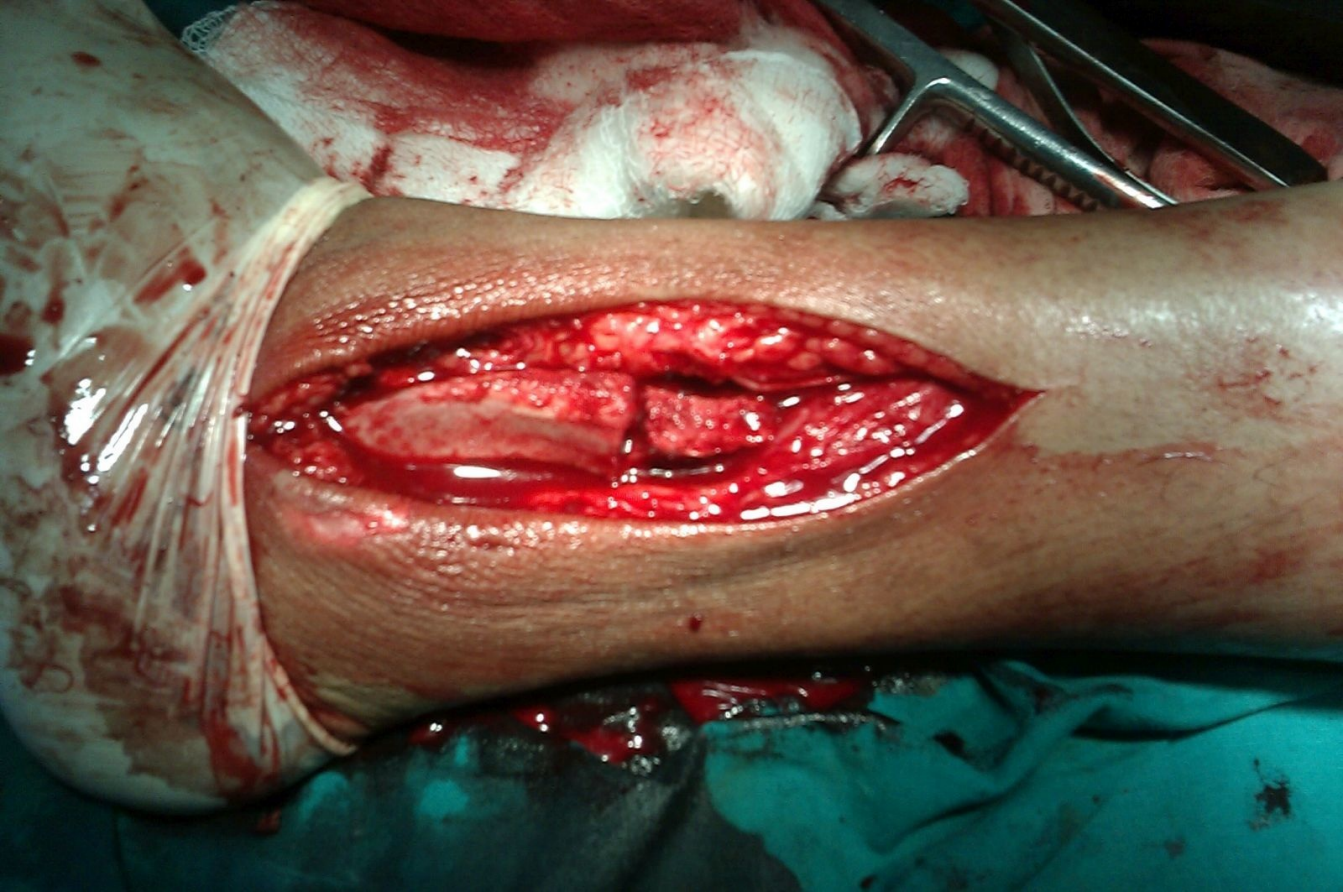
Intra-operative picture showing autologous iliac crest bone graft to reconstruct the ankle mortise

The bone defect was reconstructed using a tricortical iliac crest autograft (Figure 4) and fixation was done with a 3.5 mm (8 holes) reconstruction plate. Two screws were passed through the syndesmosis to augment it. The lateral fibular wall containing the tumour was excised, preserving the medial wall (with syndesmosis) and lateral malleolar articular surface.

**Figure 5:**
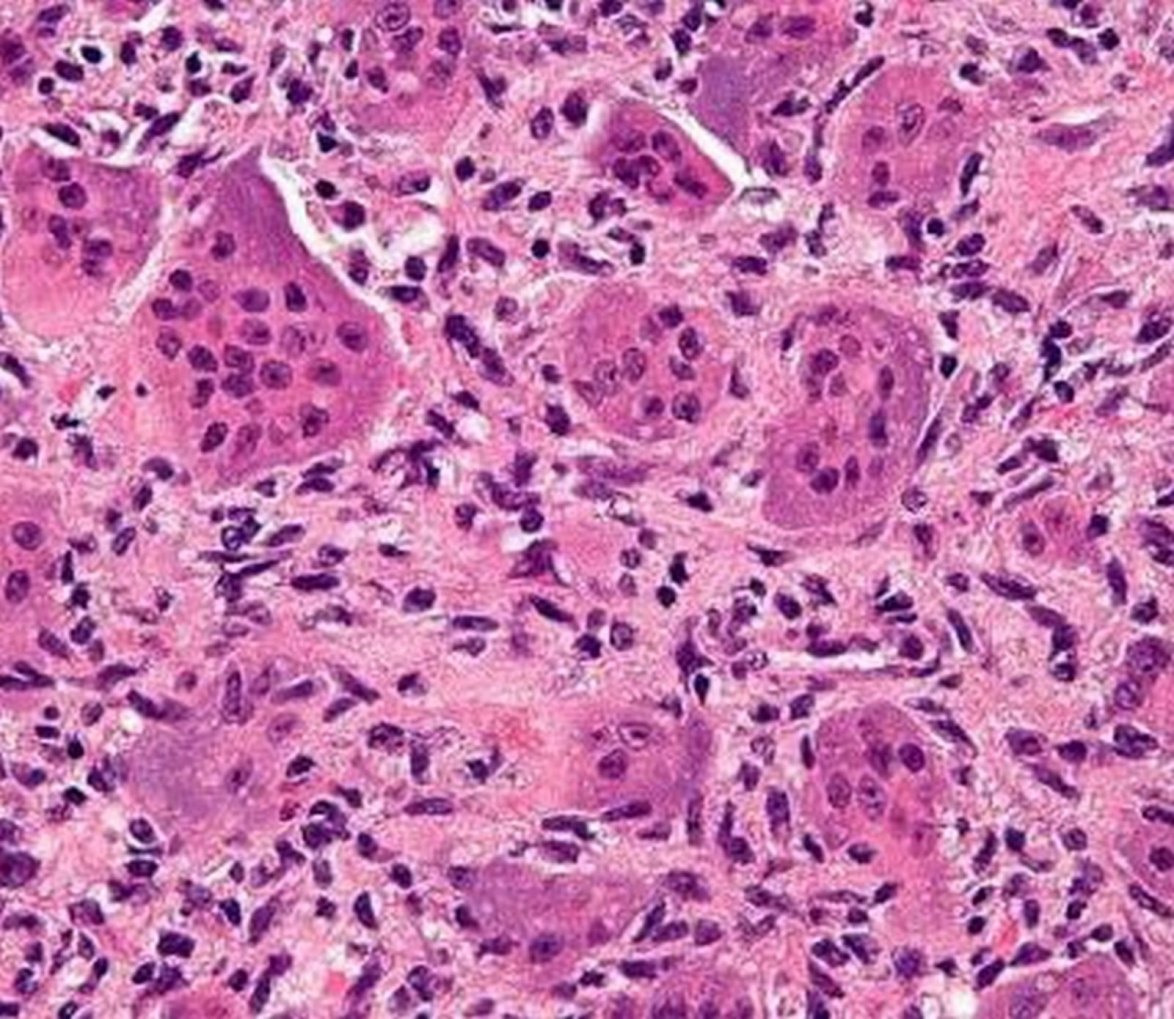
Histopathological slide showing multinucleated giant cells along with stromal cells

She was mobilized nonweight bearing, with crutches, with a below knee cast for 3 months, until the bone graft got incorporated. The histopathological features showed multinucleated giant cells (Figure 5) which were suggestive of a diagnosis of GCT. There were regular and uniform distribution of stromal cells and giant cells. The stromal cells are mononuclear, resembling macrophages while giant cells are large, multinucleated (10-50 nuclei) with similar nuclei as stromal cells, resembling osteoclasts.

**Figure 6:**
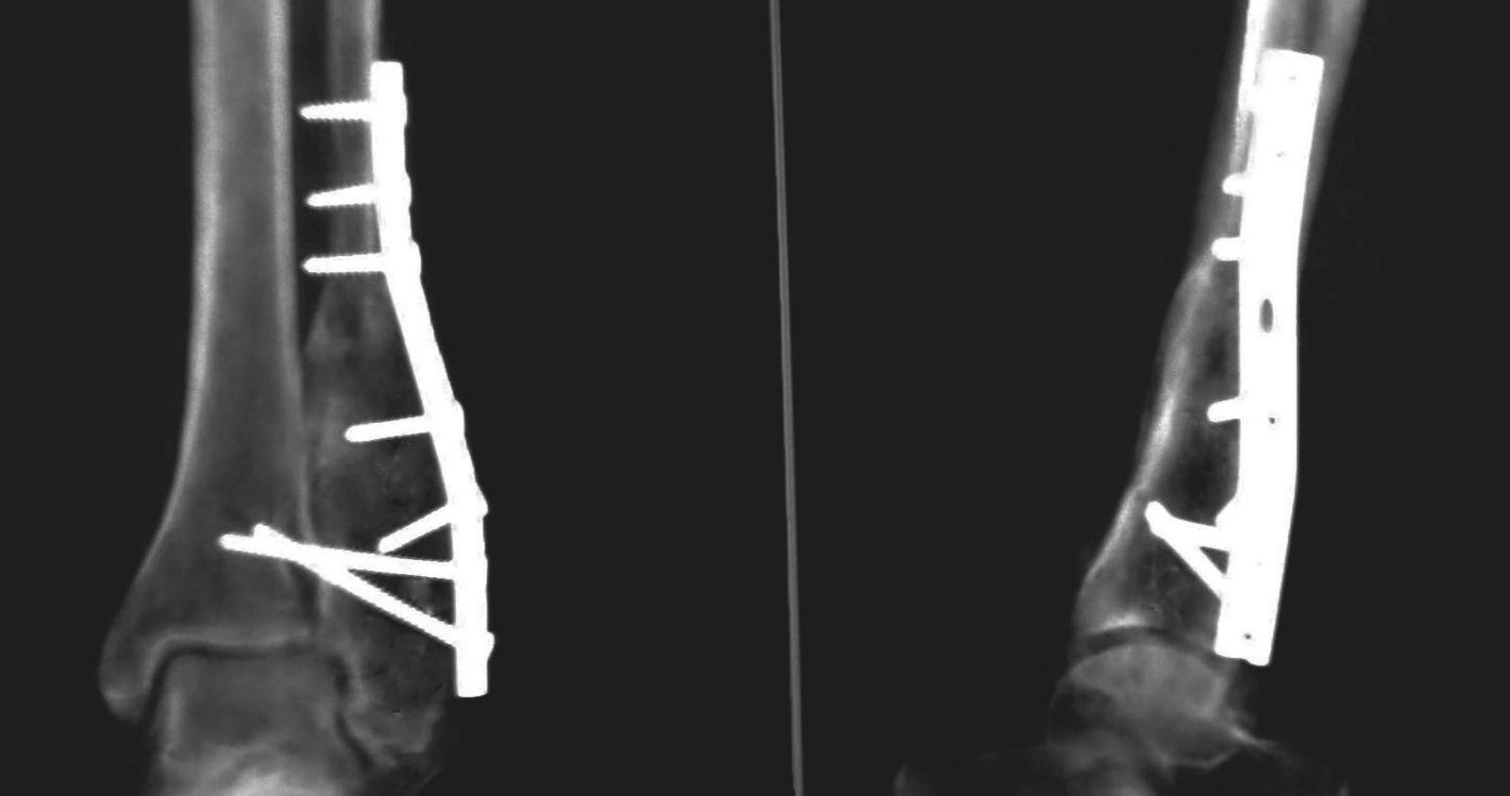
Plain radiograph (Anteroposterior and lateral view) showing 18 months follow-up case with incorporated bone graft

At 18 months follow-up, there was no evidence of local recurrence, with a full range of pain-free movements of ankle and subtalar joints (Figure 6).

## Discussion

The GCT of the distal fibula is a rare condition [3]. The treatment of aggressive GCT at this location is challenging. Only a few case reports or small case series have been published on this subject [4, 5] and hence there are no clear guidelines available for its management and outcomes.

Lower ten centimeters of fibula forms an important component for the stability of the ankle mortise. Reconstruction of lower fourth of fibula following excision for tumours is essential for stability of the ankle mortise. Hence, any lesion affecting this area needs a special consideration towards the preservation of the ankle mortise, especially in young patients with an active lifestyle. Moreover, as the deformity affects the gait, it hinders daily activities leading to a larger impact on an individual’s social and professional life.

Treatment of GCT is the surgical and intralesional excision by "extended" curettage which was the chosen procedure for this case and and the treatment of choice. The curettage is associated with a high recurrence rate if done alone. Local recurrence after curettage alone is 60 %, while recurrence after extended curettage is approximately 10-20 % [6]. The addition of chemical agents (adjuvants), helps to decrease the rate of recurrence. Various agents have been used like phenol, liquid nitrogen, bone cement, hydrogen peroxide, zinc chloride, and argon. We prefer to use phenol, because of its ready availability, ease of application, low cost, and known benefits. These chemical or physical agents work by inducing an additional circumferential area of necrosis to extend the curettage. Cryosurgery using liquid nitrogen is associated with a high incidence of a local wound and bone complications [7] and hence is not popular anymore.

Post-excision of the tumor, reconstruction of the joint surface is required as GCT invariably involves the ends of a long bone and causes a destruction of the joint surface. If the defect after the curettage is small and does not compromise the structural integrity of the bone, no graft or cement is required. The void gets filled up with blood clots which then ossify to form the bone [8]. For larger defects cementation or use of bone graft is required. The advantage of bone graft is that it undergoes remodeling, and the reconstruction is permanent once the graft gets incorporated. Reconstruction of distal fibula following excision of the GCT requires not only bone grafting but also the preservation of ankle mortise. This can be achieved by using autograft either from the fibula [9] or by using a tricortical graft from the iliac crest, like in our case. When wide excision of the distal fibula is required, the distal fibula with bone graft needs to be protected and we achieved this by using a 3.5 mm reconstruction plate.

Recurrence of the tumor usually occurs within the first two years but late ones are known as well [10]. There is an increased rate of recurrence in aggressive lesions. This could be due to the presence of residual tumour cells after a curettage, once the tumour has breached the cortex and extended into the soft tissues [8]. In this case, at 18 months of follow-up, there were no signs of recurrence and good incorporation of the iliac crest graft was observed radiologically. The stability and function of the ankle joint were also preserved well with a good range of movement. 

## Conclusions

Giant cell tumors of the distal fibula are extremely rare and pose challenges in their management. Thus, we recommend that all the aggressive and large GCT of the distal fibula must be excised adequately with adjuvant chemical cauterisation. The bony defect should be reconstructed using autogenous bone graft with internal fixation. It is mandatory to preserve the ankle mortise during reconstructive surgery, to achieve a good functional outcome.
